# COVID-19 and abducens nerve palsy in a 9-year-old girl—case report

**DOI:** 10.1186/s13052-022-01298-3

**Published:** 2022-06-18

**Authors:** Martina Capponi, Bianca Laura Cinicola, Giulia Brindisi, Cristiana Alessia Guido, Maria Cristina Torcé, Anna Maria Zicari, Alberto Spalice

**Affiliations:** 1 Department of Maternal Sciences, Sapienza University of Rome, Italy Viale Regina Elena, 324 00161 Rome, Italy; 2 Department of Molecular Medicine, Sapienza University of Rome, Rome, Italy; 3 Department of Developmental and Social Psychology, Sapienza University of Rome, Rome, Italy; 4 Department of Ophthalmology, Sandro Pertini Hospital, Rome, Italy

**Keywords:** COVID-19, Abducens nerve palsy, Diplopia, Brain MRI

## Abstract

**Background:**

Coronavirus disease 2019 (COVID-19) is a disease caused by severe acute respiratory syndrome coronavirus 2 (SARS-CoV-2) infection. Although many reports have detailed a range of neurological symptoms in SARS-CoV-2-infected patients, studies of neuro-ophthalmological manifestations are still scarce.

**Case presentation:**

We report a 9-year-old girl with abducens nerve palsy after COVID-19 with no evidence of other neurological disease on neuroimaging. At 2-month follow-up clinical conditions were improved.

**Conclusions:**

The palsy may have occurred due to a possible post-infectious immune-mediated mechanism underlying the neuropathy, as opposed to direct viral infiltration. Despite being rare, this complication must be taken into account.

## Background

Severe acute respiratory syndrome coronavirus 2 (SARS-CoV-2) was first reported in late December 2019 in Wuhan, China [1] and is the causative agent of coronavirus disease 2019 (COVID-19), declared a pandemic health emergency by the World Health Organization on March 2020.

Clinical presentation and severity of COVID-19 vary significantly from asymptomatic subjects to patients with severe and sometimes fatal disease [2].

Although the majority of patients with SARS-CoV-2 infection have fever and respiratory symptoms like dry cough, fatigue and shortness of breath, as well as myalgia and anosmia [1, 3, 4], the virus can cause a large spectrum of clinical manifestations including heart, kidney, vascular damage, impaired coagulation, and neurological injury [5].

Numerous evidences support the neurotropic and neuro-invasive potential of SARS-CoV-2 [6], which can involve the central nervous system, the peripheral nervous system, the muscle, as well as olfactory tract [7, 8].

Acute cranial nerve (CN) paresis, including abducens and oculomotor nerve involvement, have been already reported among adults and young adults, but the VI nerve palsy is not well described in children [9–15].

In this report, we describe a 9-year-old female patient who presented isolated abducens nerve palsy after COVID-19, with no evidence of other neurological disease on neuroimaging and absence of preexisting vascular risk factors.

## Case presentation

We report on a 9-year-old girl with acute onset of persistent diplopia seventeen days after a mostly asymptomatic SARS-CoV-2 infection. The main symptom reported during the course of the disease was headache, but there was no meningismus. The diagnosis was confirmed using a molecular nasopharyngeal swab that detected the presence of SARS-CoV-2 RNA by real-time reverse transcriptase polymerase chain reaction (PCR). The test resulted negative after 10 days from the beginning of the infection.

An intercurrent episode of fever (temperature 38.5 °C) lasting 72 hours was reported one week before the onset of the eye disorder. The patient’s parents noticed the sudden onset of convergent strabismus of the right eye while the child was using her mobile phone. She had no pain with eye movements or other neurologic symptoms such as weakness, ataxia, paresthesia and hyporeflexia or anosmia/ageusia.

**Fig. 1:**
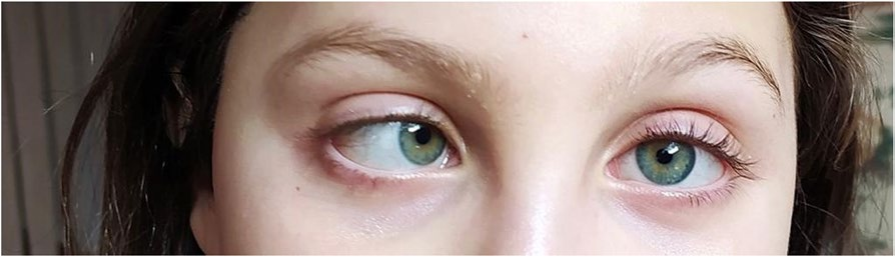
Picture of ophtalmological examination. Incomitant esotropia (convergent squint) with limitation of abduction in the right eye at ocular motility examination

The first clinical examination at the Pediatric Department Child Neurology Unit revealed complete and isolated failure of abduction of the right eye. The horizontal diplopia, persisted in the primary gaze and was exacerbated by distant fixation (Fig. 1). Ocular motility was full, the pupillary response was preserved and there was no evidence of ptosis, proptosis, chemosis or significant ocular exudate, or fatiguing weakness. The remaining neurological examination and the rest of the physical examination were unremarkable.

On ophthalmologic examination her best-corrected visual acuity was normal in both eyes, no afferent pupillary defect nor nystagmus was observed. The colour vision (Hishihara plates), slit lamp examination and visual field were normal in both eyes. Ocular motility examination revealed an incomitant esotropia (convergent squint) with limitation of abduction in the right eye and abnormal position of the head when turned to the right shoulder. Horizontal binocular vision with image separation was typically worse at long distance than at near distance, and worse for right gaze. The fundus of the eye was normal.

Magnetic resonance imaging (MRI) of the brain was performed with contrast-enhanced multiplanar reconstruction study, revealing normal findings.

Lumbar puncture showed a normal opening pressure and normal cerebro-spinal fluid (CSF) components. The search for the main neurotropic viruses including the analysis for SARS-CoV-2 RNA as well as for oligoclonal bands was negative.

Routine blood tests were normal including complete blood count, coagulation, inflammatory markers, liver and kidney function.

The patient was discharged after 48 hours of observation with no change in double vision.

After the collegial discussion of the case no treatment was undertaken.

At one week follow-up, the patient reported the persistence of diplopia with a fluctuating course. Patching of the good eye was advised and performed. At 2-month follow-up, the patient had improved binocular vision and reduced eye deviation.

## Discussion and conclusions

The aetiology of sixth nerve palsy can be congenital or acquired. The most common cause of acquired abducens palsy in children has been found to be neoplasm, unlike the more common vascular aetiology in adults [16, 17]. The second most common cause is trauma [18, 19]. Other causes include elevated intracranial pressure, congenital, inflammation, idiopathic and post-viral [20]. Although there is a close association between sixth nerve palsy and viral illness, the exact pathophysiology remains unknown [21–23].

Regarding SARS-CoV-2, while the amount of data published on central and peripheral neurological manifestations is continuously growing, few studies describe the specific neuro-ophthalmological manifestations [10]. These include headache, ocular pain, visual impairment, diplopia and cranial nerve palsies secondary to Miller Fisher syndrome (MFS), Guillain-Barré syndrome (GBS), or encephalitis and nystagmus [10, 24].

We therefore decided to report the case of a young girl with diplopia caused by VI nerve palsy, investigating the possible etiopathogenetic relationship with primary SARS-CoV-2 infection.

The neurotropism of SARS-CoV-2 [25, 26] is mediated by the human angiotensin converting enzyme 2 (hACE2) receptor, which is ubiquitously expressed and used by the virus to enter the host cell. The co-receptors and attachment factors, such as transmembrane serine protease 2 (TMPRSS2) and basigin (BSG, also known as CD147), enhance the entry of the virus in the presence of ACE2. Furthermore, neuropilin 1 (NRP1) mainly facilitates the regulation of angiogenesis, gangliogenesis and vascular permeability, and similarly enhances viral infectivity by acting as a co-receptor for cell entry.

The proposed pathogenetic mechanisms to explain SARS-CoV-2 neuro-invasiveness and neuro-virulence include:

- Direct viral involvement through retrograde or anterograde transport mechanisms from peripheral nerves to the CNS (for example, through the neuroepithelium of the olfactory nerve and olfactory bulb, via the cribriform plate).
- A route of hematogenous dissemination could provide entry into the CNS for SARS-CoV-2 by three mechanisms: 1) Transcellular migration that involves the binding of the virus to its receptors (ACE2, BSG or NRP-1) on brain microvasculature endothelial cells and subsequent crossing of the endothelial cells via transcytosis [27, 28]; 2) The Trojan horse mechanism, which involves the infection of immune cells that then bring the virus across the blood brain barrier (BBB) into the CNS [26]; or 3) A paracellular route by disrupting endothelial cell tight junctions [27, 29].
- A systemic inflammatory response (including autoinflammatory and hyperinflammatory responses) associated with the deleterious role of the cytokine storm in disrupting the BBB (which can lead to the development of encephalopathy/encephalitis, acute disseminated encephalomyelitis, seizures, altered consciousness).
- An immune-mediated para-infectious or post-infectious effect; it is believed that SARS-CoV-2 has auto-immunogenic effects mainly via molecular mimicry, leading to autoimmune neuropathies (affecting cranial and peripheral nerves), as demonstrated in cases of GBS and its variants MSF [30].

In our case, the primary infection was asymptomatic and the neurological disorder appeared after about 2 weeks, suggesting a post-infectious immune-mediated mechanism underlying the neuropathy rather than direct viral infiltration.

Additionally, besides diplopia, the neurological examination was completely negative (no strength deficit and good evoked reflexes were found) and the level of proteins in the CSF was normal, reducing the likelihood that it was a GBS variant. Even if in this case the anti-ganglioside antibodies have not been tested, the Brain MRI revealed normal findings.

In light of these considerations, virus-induced immune-mediated neuropathy may be the most likely pathogenic mechanism.

Further studies are needed on a larger population to verify the association between SARS-Cov-2 infection and neuro-ophthalmological disorders in order to clarify the exact etiopathogenesis.

## Data Availability

All data generated or analysed during this study are included in this published article.

## Abbreviations

COVID-19: Corona Virus Disease 2019
SARS-CoV-2: Severe Acute Respiratory Syndrome Coronavirus 2
CN: Cranial Nerve
PCR: Polymerase Chain Reaction
MRI: Magnetic Resonance Imaging
CSF: Cerebro-Spinal Fluid
MFS: Miller Fisher Syndrome
GBS: Guillain-Barré Syndrome
hACE2: Human angiotensin converting enzyme 2 receptor
TMPRSS2: Transmembrane serine protease 2
BSG: Basigin
NRP1: Neuropilin 1
CNS: Central nervous system
BBB: Blood brain barrier
